# Severe asthma and quality of life

**DOI:** 10.1186/s40413-017-0159-y

**Published:** 2017-08-21

**Authors:** Elham Hossny, Luis Caraballo, Thomas Casale, Yehia El-Gamal, Lanny Rosenwasser

**Affiliations:** 1 Pediatric Allergy and Immunology Unit, Children’s Hospital Ain Shams University, 40A Baghdad Street, Cairo, 11341 Egypt; 2 Institute for Immunological Research, University of Cartagena, Cartagena, Colombia; 3 Department of Internal Medicine, Division of Allergy and Immunology, Morsani College of Medicine, University of South Florida, Tampa, FL USA; 4 University of Missouri - Kansas City, School of Medicine, Kansas City, MO USA

**Keywords:** Severe asthma, Quality of life, Biologics, Adherence, Monoclonal antibodies

## Abstract

Severe asthma has a great impact on the quality of life (QOL) of patients and their families. The magnitude of this morbidity is affected by several personal factors including age. Appropriate asthma control and modifications of social roles and activities are expected to improve QOL. Biologics, primarily monoclonal antibodies, have been developed to target specific pathways and molecules important in the pathogenesis of asthma. The use of biologics has shown some promising effects on the QOL of patients with severe recalcitrant asthma. Other potential measures involve targeting risk factors and comorbidities and improving the levels of adherence to therapy. This article briefly reviews the impact of severe asthma on QOL and the potential methods to combat this morbidity including the available therapeutic biologics.

## Background

Severe and/or recalcitrant asthma is increasingly recognized as a major unmet need. Estimates vary worldwide, but generally 5 to 10% of patients are considered severe, that is, uncontrolled despite optimal pharmacologic therapy. Severe asthma has a great impact on the quality of life (QOL) of patients and their families. QOL is defined as the perception that individuals have of their position in life, in the context of the culture and system of values in which they live and in relation to their objectives, expectations, standards, and concerns [1].

QOL is an important construct for characterizing patient populations and evaluating therapeutic interventions, and this construct is not captured in other biological or clinical asthma outcome measures. None of the currently available QOL instruments is to be considered standard. Many instruments have been translated into languages other than English in order to address the global cultural differences. Some instruments do not adequately fit with age-related developmental capabilities. For instance, there are limited data on their value in the elderly, among whom there may be many confounding comorbidities. [2] In asthma, the conventional clinical outcomes address mortality and morbidity prevention and QOL assessment addresses the patients’ well being. A factor analysis that addressed the relationship between quality of life and clinical status in asthma has shown clearly that patient well-being cannot be imputed from clinical outcomes, and that it must be measured and interpreted independently [3].

An analysis of a large cohort of patients with severe or difficult-to treat asthma revealed that greater severity and numbers of asthma exacerbations were significantly associated with decreases in asthma-related QOL [4]. Symptoms and activity limitations are the two main domains that potentially affect the QOL in patients with severe asthma. Appropriate asthma control and modifications of other aspects of QOL, related to social roles and activities, are expected to improve QOL [5]. Also biological therapies are increasingly showing efficacy in terms of severe asthma control and hence living with better quality. We sought to briefly review the impact of severe asthma on QOL and the potential methods to combat this morbidity.

## The effects of severe asthma on QOL in relation to different age groups

The effect of asthma on QOL is expected to occur with different characteristics and magnitude according to several patient factors, including age. Moderate to severe asthma has led to a worse QOL as compared to mild persistent asthma; however, the personal burden of illness, as perceived by the patient, cannot be fully assessed by objective measures of disease severity; for asthma traditional clinical indices only moderately correlate with how patients feel and live daily [6].

The QOL concept represents the multidimensional perspective of asthma control. This is a better way to validate the impact of disease in the real life of patients. It may be directly assessed applying the QOL questionnaire (QOLQ) [7] or indirectly by the Asthma Control Test (ACT) [8]. The latter is not actually a QOL instrument but may give an idea about coping with the disease burden. Although correlation between these tools and GINA has been found, the QOLQ seems to be more appropriate for adjusting the treatment [9]. The asthma quality of life questionnaire (AQLQ) covers four health domains: activity limitation, symptoms, emotional function and environmental stimuli. A version adapted for children (Pediatric Asthma Quality of Life Questionnaire-Adapted, PAQLQ-A) is also available [10] and has been validated and translated to more than 40 languages. However, the application of this tool in daily practice is not easy, which has motivated several studies trying to detect those factors affecting more the QOL in children and adults. A study from Malaysia sought to determine clinical factors most related to QOL in adults with severe asthma. Symptom frequency, especially chest tightness and shortness of breath were strongly associated with impaired QOL scores. Among the domains measured, the ones most severely reduced were those relating to physical and emotional capabilities as well as general health status [11].

As a part of the TENOR study in the US, including adolescents and adults, Luskin et al. showed that asthma exacerbation frequency and severity and the number of triggers at baseline were strongly associated with patients’ asthma-related QOL. They also identified specific asthma triggers that were strongly associated with QOL and risk of future exacerbations [4]. In a study from England, a mini Asthma Quality of Life Questionnaire (mAQLQ), and Asthma Symptom Utility (ASUI) measures were significantly worse for patients suffering exacerbations (*p* < 0.001) compared to those without, however, no data about the domains were measured in this work [12]. A study from Italy found that QOL (as evaluated by AQLQ) is significantly impaired in the group of patients with dyspnea, chest tightness, and asthmatic crisis [13]. It is noteworthy that many of these studies were not performed in severe asthma and more investigations are certainly needed to outline the exact alterations in QOL of severe asthma patients.

Some psychological aspects associated with asthma also lower QOL. After controlling for gender, age, negative affect, and number of co-morbid medical problems in adults, the physical concerns factor of anxiety sensitivity (AS-Physical Concerns) significantly predicted asthma control and influenced all domains of asthma-related QOL (symptoms, activity limitations, emotional functioning, and environmental stimuli) [14]. Asthma severity also affects QOL in adolescents, as has been reported in several studies from different countries [15–18]. Personality traits were significantly related to QOL of adolescents with asthma. These relations were fully mediated by coping and symptom reporting [19]. A study from Brazil revealed that the QOL was directly related to asthma control and asthma severity in children and adolescents [20]. The same was observed in German children [21]. The negative impact on QOL is not only on children but also on their caregivers [22] and family functioning [23, 24].

Finally, it can be said that the most important clinical parameter affecting the QOL of patients with bronchial asthma is disease severity, defined both in terms of objective spirometry results and quasiobjective variables such as the frequency of unscheduled clinic visits and hospitalizations due to exacerbations [25]. On the other hand, Stucky et al., studying 2032 adults with asthma, have found that other factors involving social roles and activities emerged as the strongest predictors of asthma-specific QOL [5]. It seems that the level and seriousness of negative mood and severity of disease in asthmatics significantly impair QOL [26].

## Biologics in severe asthma

Biologics, primarily monoclonal antibodies, have been developed to target specific pathways and molecules thought to be important in the pathogenesis of asthma. In order to justify their expense (typically 15,000 to 25,000 USD/year), these agents have been evaluated for their ability to reduce asthma exacerbations providing a pharmacoeconomic justification for their use.

Due to the heterogeneity of asthma, attempts have been made to optimize the likelihood of positive clinical responses associated with biologics by better characterizing the individual patient’s phenotypic and endotypic characteristics, that is, the application of precision medicine. Two major endotypic categories of asthma have been proposed: T2-high (T2-hi) manifested by increased eosinophils in the sputum and airways of patients; and T2-low (T2-lo) manifested by increased neutrophils or a paucigranulocytic profile with normal eosinophil and neutrophil counts in the sputum and airways [27, 28].

Cytokines thought to be important in the pathogenesis of neutrophilic T2-lo asthma include interleukins (IL)-1, 8, 23 and 17 and TNF-alpha. A monoclonal antibody against IL-17 (brodalumab) has been studied in asthma, but did not show substantial benefits [29]. However, the patient population study was not enriched for the presence of airway neutrophilia. Due to adverse consequences, brodalumab clinical development for asthma has been stopped. Blocking TNF-alpha in severe asthma has been met with variable successes but an unacceptable risk-benefit ratio which has also led to a lack of further clinical development for asthma [28]. A CXC chemokine receptor 2 blocker (IL-8 antagonist) has shown no statistically significant improvements in pulmonary functions or symptom scores but a decrease in mild asthma exacerbations in a proof of concept study [30]. Overall, T2-lo asthma patients have few therapeutic options since the development of biologics for these patients lags behind those for T2-hi asthma and T2-lo asthma patients have greater resistance to steroids.

There have been a number of biologics developed to treat T2-hi asthma characterized by eosinophilic inflammation with or without antigen-specific IgE. Monoclonal antibodies directed against IgE, IL-4 and 13 and IL-5 have been extensively studied in asthma, and one anti-IgE and two anti-IL-5 monoclonal antibodies have been approved by the US FDA for the treatment of severe asthma [27, 28].

Omalizumab has been in clinical use for over 13 years. Numerous studies have shown that omalizumab reduces asthma exacerbation frequency, the need for corticosteroids, and asthma symptoms while improving QOL. However, omalizumab’s effects on pulmonary functions are not insistently clinically important [27, 28]. Recent data suggest that patients with higher blood eosinophil levels may respond better to omalizumab [31–33].

Mepolizumab and reslizumab, anti-IL-5 monoclonal antibodies, have recently been approved for asthma therapy. Both of these monoclonal antibodies reduced asthma exacerbations in patients with higher blood eosinophil levels. Mepolizumab has also been shown to improve QOL and FEV1 in patients with higher blood eosinophil levels as well as reducing steroid need [34–36]. Reslizumab has consistently demonstrated ability to improve FEV1 and improve QOL in patients with asthma [37–39]. Mepolizumab is dosed monthly subcutaneously whereas reslizumab is dosed monthly intravenously.

Benralizumab has a different mechanism of action. It binds to the IL-5 receptor and causes antibody-dependent cell-mediated cytotoxicity of both eosinophils and basophils. It has been shown to decrease asthma exacerbation rates and improve FEV1 in severe asthma patients with elevated blood eosinophil levels [40, 41]. Benralizumab dosing has not been definitively established but will likely be subcutaneous monthly for the first three doses and then administered every eight weeks. None of the anti-IL-5 monoclonal antibodies has been shown to have clinical benefits in patients with low eosinophil counts in the peripheral blood.

Lebrikizumab and Tralokinumab are two anti-IL-13 monoclonal antibodies that have been studied for asthma. Although initially showing promise in patients with elevated blood periostin levels, replicating phase 3 studies of Lebrikizumab in severe asthma showed disparate results which has led to a lack of further clinical development for asthma [42]. Tralokinumab has been shown to have some positive results in reducing asthma exacerbations in patients with elevated levels of periostin or the dipeptidyl peptidase DPP-4 [43, 44].

Initial attempts to block IL-4 alone were unsuccessful for the management of asthma. Dupilumab is a monoclonal antibody directed against IL-4 alpha receptor, which is common to signaling for both IL-4 and IL-13. This monoclonal antibody is delivered subcutaneously every one to two weeks and has been shown to reduce asthma exacerbations and improve FEV1 and QOL in patients with severe asthma regardless of blood eosinophil levels [45, 46]. However, better effects are noted in patients with higher blood eosinophil levels.

Table 1 summarizes some of the most important characteristics of T2-hi biologics currently in clinical development or approved for the treatment of asthma and Table 2 displays data from clinical trials on the effect of some biologics on HRQOL in severe asthma patients.

**Table 1:** Biologics currently in development or approved for the treatment of asthma

Drug	Target	Biomarkers	Effects	Route/Dose
Omalizumab	IgE	Ag-specific IgE Better responses with higher FeNO and blood eosinophils >300 cells/ uL	Decreased asthma exacerbations	150–375 mg SC q2-q4wks; frequency based on IgE and body weight
Mepolizumab	IL-5	Blood eosinophil count >150 cells/uL at initiation or 300 cells/uL in past year	Decreased asthma exacerbations; Improvement in FEV1	100 mg SC q4wk.
Reslizumab	IL-5	Blood eosinophil count >400 cells/uL	Decreased asthma exacerbations; Improvement in FEV1	3 mg/kg IV q4wks
Benralizumab	IL-5Rα	Blood eosinophil count >300 cells/ uL	Decreased asthma exacerbations; Improvement in FEV1.	30 mg SC q4wks ×3 then q8wks
Tralokinumab	IL-13	Elevated blood periostin and DDP-4	Improvement in FEV1	Not Defined
Dupilumab	IL-4Rα	Better responses with blood eosinophil count >300 cells/ uL	Decreased asthma exacerbations; Improvement in FEV1	200 or 300 mg SC q2wks; administered at home

*DDP-4* dipeptidyl peptidase-4, *FeNo* fractional excretion of nitric oxide, *FEV1* forced expiratory volume in 1 s

**Table 2:** Data on impact of some biologic therapies on HRQOL of severe asthma patients

Medication	Publication	Study design	Population	Results
Omalizumab	Holgate et al., 2004 [47]	RDBPC study	Patients ≥12 years with severe allergic asthma (*n* = 246)	Improved AQLQ scores through 32 weeks.
	Humbert et al., 2005 [48]	RDBPC study	Patients ≥12 years with uncontrolled severe persistent allergic asthma (*n* = 419)	Improved AQLQ scores after 28 weeks or discontinuation.
	Hanania et al., 2011 [49]	Prospective, MC, RDBPC, parallel-group, study	Patients ≥12 years with uncontrolled severe allergic asthma (*n* = 850)	Improved mean AQLQ scores (0.29 point [CI, 0.15 to 0.43]) after 48 weeks
	Brodlie et al., 2012 [50]	16-week therapeutic study	Children with severe asthma (median age 12 years; 15 children <12 years and 19 ≥ 12 years).	Mini-AQLQ score increased from 3.5 to 5.9 (*p* < 0.0001).
	Barnes et al., 2013 [51]	MC retrospective observational study	Patients aged ≥12 years with severe persistent allergic asthma (*n* = 136)	Improved median AQLQ scores at 16 weeks and up to 12 months post-omalizumab initiation.
	Braunstahl et al., 2013 [52]	International, single-arm, open-label, observational registry	Uncontrolled persistent allergic asthma (*n* = 943). Two patients aged <12; the rest ≥12 years.	Clinically relevant (> 0.5 point) improvement from baseline in the AQLQ and mini-AQLQ scores in 67.2% of patients at month 12 and 60.7% at month 24.
	Odajima et al., 2015 [53]	multicenter, uncontrolled, open label study	Children (6–15 years) with uncontrolled severe allergic asthma (*n* = 38)	Improved scores of asthma-specific QOL questionnaire for pediatric patients after 24 weeks.
	Li et al., 2016 [54]	RDBPC, parallel-group, phase III study	Patients ≥18 years with uncontrolled moderate-to-severe persistent allergic asthma (*n* = 616)	Improved overall AQLQ scores and all individual domain scores after 24 weeks.
	Alhossan et al., 2017 [55]	Meta-analysis (24 observational studies across 32 countries)	Adults with Severe Allergic Asthma (*n* = 9213)	Improvements in quality of life at 4–6 months (Cohen’s d = 1.05; 1.29 AQLQ points) and at 12 months of therapy (Cohen’s d = 1.20; 1.51 AQLQ points).
Mepolizumab	Haldar et al., 2009 [56]	RDBPC, parallel group trial	Adults with refractory eosinophilic asthma (*n* = 61)	Improved AQLQ scores along one year (mean increase from baseline 0.55)
	Ortega et al., 2014 [33]	RDBPC, double dummy study	Adults with severe eosinophilic asthma (*n* = 576)	Improved SGRQ scores after 32 weeks
	Bel et al., 2014 [35]	RDBPC study	Adults with severe eosinophilic asthma (*n* = 135)	Improved SGRQ scores after 24 weeks
	Magnan et al., 2016 [57]	Post hoc analyses from two RDBPC, parallel group, studies	Adults with severe eosinophilic asthma previously treated with omalizumab (*n* = 711)	Improved SGRQ (after 24–32 weeks) vs placebo in both studies independent of prior omalizumab use.
	Chupp et al., 2017 [58]	RDBPC, MC, parallel-group, phase 3b study	Patients ≥12 years with severe eosinophilic asthma (*n* = 551)	A significant improvement in HRQOL from baseline (SGRQ total score at week 24) and a safety profile similar to that of placebo.
Lebrikizumab	Hanania et al., 2015 [59]	Pooled data from two MC, RDBPC studies	Patients ≥18 years with uncontrolled moderate-severe asthma (*n* = 463)	No placebo-corrected improvements in AQLQ scores at week 12 (wide confidence intervals) despite improvements in lung functions.
Tralokinumab	Piper et al., 2013 [42]	RDBPC, MC, parallel-group, phase 2a study	Patients >18 years with uncontrolled moderate-severe asthma (*n* = 194)	No differential effect was apparent in any of the patient reported outcomes at week 12 despite improved lung functions.
	Brightling et al., 2015 [43]	RDBPC, MC, parallel-group, phase 2b study	Patients >18 years with uncontrolled severe asthma (*n* = 383)	Improvement in AQLQ[S] (*p* = 0.019) in patients treated every 2 weeks (*n* = 33) compared with placebo (*n* = 48)

*AQLQ* Asthma Quality of Life Questionnaire, *HRQOL* health related quality of life, *MC* multicentre, *RDBPC* randomized double-blind placebo-controlled, *SGRQ* St George’s Respiratory Questionnaire

## Other potential measures to improve QOL in severe asthma

Adherence to therapy is a determinant of improved asthma control and hence better QOL in severe asthma. A few adherence interventions have been studied closely in asthma. These include shared decision-making for medication and dose choice, inhaler reminders for missed doses, reduced complexity of the regimen (once versus twice daily), comprehensive asthma education with home visits by asthma nurses and clinicians reviewing feedback on their patients’ dispensing records [60].

Asthma education can lower risk of future emergency department visits and hospital admission [61]. Asthma education should highlight the importance of adherence to prescribed inhaled corticosteroids (ICS) even in the absence of symptoms [62]. This mandates that asthma education follow a repetitive pattern and involve literal explanation and physical demonstration of the optimal use of inhaler devices and should be tailored according to the socio-cultural background of the family. Inclusion of interactive components such as workshops, video games, internet programs [63], art therapy group sessions [64], and telephone asthma coaching [65] were reported to improve asthma control and hence QOL.

Targeted parenting skills were chosen to address treatment resistance in a prospective study. After the 6-month intervention, adherence with inhaled corticosteroids increased from 72.9 to 100.0%, (*p* = 0.013). The percentage of children with controlled asthma increased from 0 to 62.5% (*p* = 0.026) indicating a clinically meaningful change. Parents’ ratings at 6 months suggested that asthma-related tasks and child behaviors were less problematic and their confidence to manage asthma increased [66]. Interventions designed to improve family functioning may actually reduce the extent to which children are distressed by their symptoms [67].

It was suggested that once-daily ICS therapy provides a practical therapeutic option that did not appear to jeopardize the clinical efficacy of asthma controller therapy. [68] Once-daily dosing strategy was associated with lower costs and higher level of quality-adjusted life-years (QALYs) [69].

Patients with asthma are encouraged to engage in sports and physical activities to achieve general well being, reduce cardiovascular risk, and improve QOL (evidence A). However, it does not confer specific benefit on lung functions or asthma symptoms per se with the exception of swimming in young patients (evidence B). Exercise-induced asthma can always be reduced by maintenance ICS and the use of SABA before or during exercise [60]. It was demonstrated that aerobic exercise reduces nuclear factor kappa light-chain enhancer of activated B cells (NF-κB) activation and increases release of the anti-inflammatory cytokine interleukin (IL)-10 [70]. Low- to moderate-intensity aerobic exercise was found to reduce asthmatic inflammation in clinical and experimental models [71].

Some risk factors contribute to severe asthma and alter the QOL including the presence of GERD, chronic rhinosinusitis, obesity, and confirmed food allergy. Weight reduction of even 5–10% can lead to better asthma control and QOL (Evidence B) [60]. Risk ratio analysis showed that obese children had a higher likelihood of going to the emergency department and of hospitalization than the overweight and normal-weight groups [72]. Older male children with more severe asthma who had at least one smoking parent reported lower asthma-specific QOL according to self- and proxy reports. [73]. Symptoms of gastroesophageal dysmotility are an independent predictor of cough-specific QOL of patients with cough variant asthma [74].

Panic disorder is a common anxiety disorder among asthmatic patients with overlapping symptoms (e.g., hyperventilation). It is associated with poor asthma control and QOL and may thus be an important target for treatment [75]. Sleep disturbances, such as difficulty initiating and maintaining sleep and early morning awakenings, are commonly reported by patients with asthma [76]. Sleep quality, independent of gastroesophageal reflux disease and obstructive sleep apnea has been associated with worse asthma control and QOL in patients with asthma, even after controlling for relevant covariates. Future research is warranted to determine if behavioral and/or medical treatment of sleep problems can improve asthma control and QOL [77].

A multicenter observational study suggested that temperature-controlled laminar airflow as a non-pharmacological management approach significantly reduced allergen exposure and airway inflammation and improved the QOL of patients with poorly controlled allergic asthma [78]. Another treatment option that was suggested for patients with severe therapy-refractory asthma is bronchial thermoplasty. It is said to down-regulate selectively structural abnormalities involved in airway narrowing and bronchial reactivity, particularly airway smooth muscles, neuroendocrine epithelial cells, and bronchial nerve endings [79]. The overall quality of evidence regarding this procedure is moderate and it leads to a modest clinical benefit in QOL [80].

## Conclusions

In conclusion, severe asthma is detrimental to the quality of life of patients. Therapies targeted to improve QOL include the approved and emerging biologics as well as combating risk factors and comorbidities, and improving the levels of disease control.

## Key Notes

- The most important clinical parameter affecting the QOL of patients with bronchial asthma is disease severity.
- A number of biologics have been developed to treat asthma characterized by eosinophilic inflammation with or without antigen-specific IgE.
- An anti-IgE and two anti-IL-5 monoclonal antibodies are approved for the treatment of severe asthma and were correlated with better QOL in several trials.
- QOL in severe asthma could also be improved by achieving better adherence to therapy, potentiating health education, addressing risk factors, and targeting social and psychological domains

## Abbreviations

ACT: Asthma control test
AQLQ: Asthma quality of life questionnaire
ASUI: Asthma symptom utility
DDP-4: Dipeptidyl peptidase-4
FeNo: Fractional excretion of nitric oxide
FEV1: Forced expiratory volume in 1 s
GERD: Gastroesophageal regurgitation disease
GINA: Global initiative for asthma
HRQOL: Health related quality of life
ICS: Inhaled corticosteroids
mAQLQ: Mini asthma quality of life questionnaire
MC: Multicenter
PAQLQ-A: Pediatric asthma quality of life questionnaire-adapted
QALYs: Quality-adjusted life-years
QOLQ: Quality of life questionnaire
RDBPC: Randomized double-blind placebo-controlled
SGRQ: St George’s respiratory questionnaire
TENOR: The epidemiology and natural history of asthma: outcomes and treatment regimens

## References

[1] The World Health Organization Quality of Life assessment (WHOQOL): position paper from the World Health Organization. Soc Sci Med. 1995;41(10):1403–9. 10.1016/0277-9536(95)00112-k 8560308

[2] Wilson SR, Rand CS, Cabana MD, Foggs MB, Halterman JS, Olson L, Vollmer WM, Wright RJ, Taggart V (2012). Asthma outcomes: quality of life. J Allergy Clin Immunol.

[3] Juniper EF, Wisniewski ME, Cox FM, Emmett AH, Nielsen KE, O'Byrne PM (2004). Relationship between quality of life and clinical status in asthma: a factor analysis. Eur Respir J.

[4] Luskin AT, Chipps BE, Rasouliyan L, Miller DP, Haselkorn T, Dorenbaum A (2014). Impact of asthma exacerbations and asthma triggers on asthma-related quality of life in patients with severe or difficult-to-treat asthma. J Allergy Clin Immunol Pract.

[5] Stucky BD, Sherbourne CD, Edelen MO, Eberhart NK (2015). Understanding asthma-specific quality of life: moving beyond asthma symptoms and severity. Eur Respir J.

[6] Moy ML, Israel E, Weiss ST, Juniper EF, Dubé L, Drazen JM, NHBLI Asthma Clinical Research Network (2001). Clinical predictors of health-related quality of life depend on asthma severity. Am J Respir Crit Care Med.

[7] Juniper EF, Guyatt GH, Epstein RS, Ferrie PJ, Jaeschke R, Hiller TK (1992). Evaluation of impairment of health related quality of life in asthma: development of a questionnaire for use in clinical trials. Thorax.

[8] Nathan RA, Sorkness CA, Kosinski M, Schatz M, Li JT, Marcus P, Murray JJ, Pendergraft TB (2004). Development of the asthma control test: a survey for assessing asthma control. J Allergy Clin Immunol.

[9] Alpaydin AO, Bora M, Yorgancioglu A, Coskun AS, Celik P (2012). Asthma control test and asthma quality of life questionnaire association in adults. Iran J Allergy Asthma Immunol.

[10] Juniper EF, Guyatt GH, Feeny DH, Ferrie PJ, Griffith LE, Townsend M (1996). Measuring quality of life in children with asthma. Qual Life Res.

[11] Hooi LN (2003). What are the clinical factors that affect quality of life in adult asthmatics?. Med J Malaysia.

[12] Lloyd A, Price D, Brown R (2007). The impact of asthma exacerbations on health-related quality of life in moderate to severe asthma patients in the UK. Prim Care Respir J.

[13] Riccioni G, D'Orazio N, Di Ilio C, Menna V, Guagnano MT, Della VR (2004). Quality of Life and clinical symptoms in asthmatic subjects. J Asthma.

[14] Avallone KM, McLeish AC, Luberto CM, Bernstein JA (2012). Anxiety sensitivity, asthma control, and quality of life in adults with asthma. J Asthma.

[15] Altiparmak S, Altiparmak O, Sari HY (2011). Asthma and quality of life in adolescents in Manisa, Turkey. Int J Adolesc Med Health.

[16] Sundell K, Bergström SE, Hedlin G, Ygge BM, Tunsäter A (2011). Quality of life in adolescents with asthma, during the transition period from child to adult. Clin Respir J.

[17] Matterne U, Schmitt J, Diepgen TL, Apfelbacher C (2011). Children and adolescents’ health-related quality of life in relation to eczema, asthma and hay fever: results from a population-based cross-sectional study. Qual Life Res.

[18] Svavarsdottir EK, Burkhart PV, Rayens MK, Orlygsdottir B, Oakley MG (2011). Icelandic and United States families of adolescents with asthma: predictors of health-related quality of life from the parents’ perspective. J Clin Nurs.

[19] Van De Ven MO, Engels RC (2011). Quality of life of adolescents with asthma: the role of personality, coping strategies, and symptom reporting. J Psychosom Res.

[20] Matsunaga NY, Ribeiro MA, Saad IA, Morcillo AM, Ribeiro JD, Toro AA (2015). Evaluation of quality of life according to asthma control and asthma severity in children and adolescents. J Bras Pneumol.

[21] Goldbeck L, Koffmane K, Lecheler J, Thiessen K, Fegert JM (2007). Disease severity, mental health, and quality of life of children and adolescents with asthma. Pediatr Pulmonol.

[22] Cerdan NS, Alpert PT, Moonie S, Cyrkiel D, Rue S (2012). Asthma severity in children and the quality of life of their parents. Appl Nurs Res.

[23] Sawyer MG, Spurrier N, Whaites L, Kennedy D, Martin AJ, Baghurst P (2000). The relationship between asthma severity, family functioning and the health-related quality of life of children with asthma. Qual Life Res.

[24] Ungar WJ, Macdonald T, Cousins M (2005). Better breathing or better living? A qualitative analysis of the impact of asthma medication acquisition on standard of living and quality of life in low-income families of children with asthma. J Pediatr Health Care.

[25] Uchmanowicz B, Panaszek B, Uchmanowicz I, Rosińczuk J (2016). Clinical factors affecting quality of life of patients with asthma. Patient Prefer Adherence.

[26] Ekici A, Ekici M, Kara T, Keles H, Kocyigit P (2006). Negative mood and quality of life in patients with asthma. Qual Life Res.

[27] Muraro A, Lemanske RF, Hellings PW, Akdis CA, Bieber T, Casale TB, Jutel M, Ong PY, Poulsen LK, Schmid-Grendelmeier P, Simon HU, Seys SF, Agache I (2016). Precision medicine in patients with allergic diseases: airway diseases and atopic dermatitis-PRACTALL document of the European Academy of Allergy and Clinical Immunology and the American Academy of Allergy, Asthma and Immunology. J Allergy Clin Immunol.

[28] Stokes JR, Casale TB (2016). Characterization of asthma endotypes: implications for therapy. Ann Allergy Asthma Immunol.

[29] Busse WW, Holgate S, Kerwin E, Chon Y, Feng J, Lin J, Lin SL (2013). Randomized, double-blind, placebo-controlled study of brodalumab, a human anti-IL-17 receptor monoclonal antibody, in moderate to severe asthma. Am J Respir Crit Care Med.

[30] Nair P, Gaga M, Zervas E, Alagha K, Hargreave FE, O'Byrne PM, Stryszak P, Gann L, Sadeh J, Chanez P, Study Investigators (2012). Safety and efficacy of a CXCR2 antagonist in patients with severe asthma and sputum neutrophils: a randomized, placebo-controlled clinical trial. Clin Exp Allergy.

[31] Hanania NA, Wenzel S, Rosén K, Hsieh HJ, Mosesova S, Choy DF, Lal P, Arron JR, Harris JM, Busse W (2013). Exploring the effects of omalizumab in allergic asthma: an analysis of biomarkers in the EXTRA study. Am J Respir Crit Care Med.

[32] Busse W, Spector S, Rosen K, Wang Y, Alpan O (2013). High eosinophil count: a potential biomarker for assessing successful omalizumab treatment effects. J Allergy Clin Immunol.

[33] Casale TB, Omachi TA, Trzaskoma B, Rao S, Chou W, Ortiz B, Manga V, Djukanovic R (2015). Estimated asthma exacerbation reduction from omalizumab in a severe eosinophilic asthma population [abstract]. J Allergy Clin Immunol.

[34] Ortega HG, Liu MC, Pavord ID, Brusselle GG, FitzGerald JM, Chetta A, Humbert M, Katz LE, Keene ON, Yancey SW, Chanez P, MENSA Investigators (2014). Mepolizumab treatment in patients with severe eosinophilic asthma. N Engl J Med.

[35] Pavord ID, Korn S, Howarth P, Bleecker ER, Buhl R, Keene ON, Ortega H, Chanez P (2012). Mepolizumab for severe eosinophilic asthma (DREAM): a multicentre, double-blind, placebo-controlled trial. Lancet.

[36] Bel EH, Wenzel SE, Thompson PJ, Prazma CM, Keene ON, Yancey SW, Ortega HG (2014). Pavord ID; SIRIUS Investigators. Oral glucocorticoid-sparing effect of mepolizumab in eosinophilic asthma. N Engl J Med.

[37] Castro M, Zangrilli J, Wechsler ME, Bateman ED, Brusselle GG, Bardin P, Murphy K, Maspero JF, O'Brien C, Korn S (2015). Reslizumab for inadequately controlled asthma with elevated blood eosinophil counts: results from two multicentre, parallel, double-blind, randomised, placebo-controlled, phase 3 trials. Lancet Respir Med.

[38] Corren J, Weinstein S, Janka L, Zangrilli J, Garin M (2016). Phase 3 study of reslizumab in patients with poorly controlled asthma: effects across a broad range of eosinophil counts. Chest.

[39] Bjermer L, Lemiere C, Maspero J, Weiss S, Zangrilli J, Germinaro M (2016). Reslizumab for inadequately controlled asthma with elevated blood eosinophil levels: a randomized phase 3 study. Chest.

[40] Bleecker ER, FitzGerald JM, Chanez P, Papi A, Weinstein SF, Barker P, Sproule S, Gilmartin G, Aurivillius M, Werkström V, Goldman M, SIROCCO study investigators. Efficacy and safety of benralizumab for patients with severe asthma uncontrolled with high-dosage inhaled corticosteroids and long-acting β(2)-agonists (SIROCCO): a randomised, multicentre, placebo-controlled phase 3 trial. Lancet. 2016;388(10056):2115-27. 10.1016/S0140-6736(16)31324-1 27609408

[41] FitzGerald JM, Bleecker ER, Nair P, Korn S, Ohta K, Lommatzsch M, Ferguson GT, Busse WW, Barker P, Sproule S, Gilmartin G, Werkström V, Aurivillius M, Goldman M, CALIMA study investigators (2016). Benralizumab, an anti-interleukin-5 receptor α monoclonal antibody, as add-on treatment for patients with severe, uncontrolled, eosinophilic asthma (CALIMA): a randomised, double-blind, placebo-controlled phase 3 trial. Lancet.

[42] Hanania NA, Korenblat P, Chapman KR, Bateman ED, Kopecky P, Paggiaro P, Yokoyama A, Olsson J, Gray S, Holweg CT, Eisner M, Asare C, Fischer SK, Peng K, Putnam WS, Matthews JG (2016). Efficacy and safety of lebrikizumab in patients with uncontrolled asthma (LAVOLTA I and LAVOLTA II): replicate, phase 3, randomised, double-blind, placebo-controlled trials. Lancet Respir Med.

[43] Piper E, Brightling C, Niven R, Oh C, Faggioni R, Poon K, She D, Kell C, May RD, Geba GP, Molfino NA (2013). A phase II placebo-controlled study of tralokinumab in moderate-to-severe asthma. Eur Respir J.

[44] Brightling CE, Chanez P, Leigh R, O'Byrne PM, Korn S, She D, May RD, StreicherK RK, Piper E (2015). Efficacy and safety of tralokinumab in patients with severe uncontrolled asthma: a randomised, double-blind, placebo-controlled, phase 2b trial. Lancet Respir Med.

[45] Wenzel S, Ford L, Pearlman D, Spector S, Sher L, Skobieranda F, Wang L, Kirkesseli S, Rocklin R, Bock B, Hamilton J, Ming JE, Radin A, Stahl N, Yancopoulos GD, Graham N, Pirozzi G (2013). Dupilumab in persistent asthma with elevated eosinophil levels. N Engl J Med.

[46] Wenzel S, Castro M, Corren J, Maspero J, Wang L, Zhang B, Pirozzi G, Sutherland ER, Evans RR, Joish VN, Eckert L, Graham NM, Stahl N, Yancopoulos GD, Louis-Tisserand M, Teper A (2016). Dupilumab efficacy and safety in adults with uncontrolled persistent asthma despite use of medium-to-high-dose inhaled corticosteroids plus a long-acting β2 agonist: a randomised double-blind placebo-controlled pivotal phase 2b dose-ranging trial. Lancet.

[47] Holgate ST, Chuchalin AG, Hebert J, Lotvall J, Persson GB, Chung KF, Bousquet J, Kerstjens HA, Fox H, Thirlwell J, Cioppa GD, Omalizumab 011 International Study Group (2004). Efficacy and safety of a recombinant anti-immunoglobulin E antibody (omalizumab) in severe allergic asthma. Clin Exp Allergy.

[48] Humbert M, Beasley R, Ayres J, Slavin R, Hébert J, Bousquet J, Beeh KM, Ramos S, Canonica GW, Hedgecock S, Fox H, Blogg M, Surrey K (2005). Benefits of omalizumab as add-on therapy in patients with severe persistent asthma who are inadequately controlled despite best available therapy (GINA 2002 step 4 treatment): INNOVATE. Allergy.

[49] Hanania NA, Alpan O, Hamilos DL, Condemi JJ, Reyes-Rivera I, Zhu J, Rosen KE, Eisner MD, Wong DA, Busse W (2011). Omalizumab in severe allergic asthma inadequately controlled with standard therapy: a randomized trial. Ann Intern Med.

[50] Brodlie M, McKean MC, Moss S, Spencer DA (2012). The oral corticosteroid-sparing effect of omalizumab in children with severe asthma. Arch Dis Child.

[51] Barnes N, Menzies-Gow A, Mansur AH, Spencer D, Percival F, Radwan A, Niven R (2013). Effectiveness of omalizumab in severe allergic asthma: a retrospective UK real-world study. J Asthma.

[52] Braunstahl GJ, Chen CW, Maykut R, Georgiou P, Peachey G, Bruce J (2013). The eXpeRience registry: the ‘real-world’ effectiveness of omalizumab in allergic asthma. Respir Med.

[53] Odajima H, Ebisawa M, Nagakura T, Fujisawa T, Akasawa A, Ito K, Doi S, Yamaguchi K, Katsunuma T, Kurihara K, Kondo N, Sugai K, Nambu M, Hoshioka A, Yoshihara S, Sato N, Seko N, Nishima S (2015). Omalizumab in Japanese children with severe allergic asthma uncontrolled with standard therapy. Allergol Int.

[54] Li J, Kang J, Wang C, Yang J, Wang L, Kottakis I, Humphries M (2016). Zhong N; China Omalizumab Study Group. Omalizumab improves quality of life and asthma control in Chinese patients with moderate to severe asthma: a randomized phase III study. Allergy Asthma Immunol Res.

[55] Alhossan A, Lee CS, MacDonald K, Abraham I. “Real-life” effectiveness studies of omalizumab in adult patients with severe allergic asthma: Meta-analysis. J Allergy Clin Immunol Pract. 2017;25 [Epub ahead of print] 10.1016/j.jaip.2017.02.002 28351783

[56] Haldar P, Brightling CE, Hargadon B, Gupta S, Monteiro W, Sousa A, Marshall RP, Bradding P, Green RH, Wardlaw AJ, Pavord ID (2009). Mepolizumab and exacerbations of refractory eosinophilic asthma. N Engl J Med.

[57] Magnan A, Bourdin A, Prazma CM, Albers FC, Price RG, Yancey SW, Ortega H (2016). Treatment response with mepolizumab in severe eosinophilic asthma patients with previous omalizumab treatment. Allergy.

[58] Chupp GL, Bradford ES, Albers FC, Bratton DJ, Wang-Jairaj J, Nelsen LM, Trevor JL, Magnan A, Ten Brinke A (2017). Efficacy of mepolizumab add-on therapy on health-related quality of life and markers of asthma control in severe eosinophilic asthma (MUSCA): a randomised, double-blind, placebo-controlled, parallel-group, multicentre, phase 3b trial. Lancet Respir Med.

[59] Hanania NA, Noonan M, Corren J, Korenblat P, Zheng Y, Fischer SK, Cheu M, Putnam WS, Murray E, Scheerens H, Holweg CT, Maciuca R, Gray S, Doyle R, McClintock D, Olsson J, Matthews JG, Yen K (2015). Lebrikizumab in moderate-to-severe asthma: pooled data from two randomised placebo-controlled studies. Thorax.

[60] Global Initiative for Asthma. Pocket Guide for Asthma Management and Prevention. Updated April 2016. Available at: http://ginasthma.org/wp-content/uploads/2016/01/GINA_Pocket_2015.pdf. Accessed 20 Nov 2016.

[61] Boyd M, Lasserson TJ, McKean MC, Gibson PG, Ducharme FM, Haby M (2009). Interventions for educating children who are at risk of asthma related emergency department attendance. Cochrane Database Syst Rev.

[62] Andrade WC, Camargos P, Lasmar L, Bousquet J (2010). A pediatric asthma management program in a low-income setting resulting in reduced use of health service for acute asthma. Allergy.

[63] McWhirter J, McCann D, Coleman H, Calvert M, Warner J (2008). Can schools promote the health of children with asthma?. Health Educ Res.

[64] Beebe A, Gelfand EW, Bender B (2010). A randomized trial to test the effectiveness of art therapy for children with asthma. J Allergy Clin Immunol.

[65] Garbutt JM, Banister C, Highstein G, Sterkel R, Epstein J, Bruns J, Swerczek L, Wells S, Waterman B, Strunk RC, Bloomberg GR (2010). Telephone coaching for parents of children with asthma: Impact and lesson learned. Arch Pediatr Adolesc Med.

[66] Garbutt JM, Sylvia S, Rook S, Schmandt M, Ruby-Ziegler C, Luby J, Strunk RC (2015). Peer training to improve parenting and childhood asthma management skills: a pilot study. Ann Allergy Asthma Immunol.

[67] Sawyer MG, Spurrier N, Kennedy D, Martin J (2001). The relationship between the quality of life of children with asthma and family functioning. J Asthma.

[68] Wells KE, Peterson EL, Ahmedani BK, Williams LK (2013). Real-world effects of once vs greater daily inhaled corticosteroid dosing on medication adherence. Ann Allergy Asthma Immunol.

[69] Rodriguez-Martinez CE, Sossa-Briceño MP, Castro-Rodriguez JA (2016). Cost-utility analysis of once-daily versus twice-daily inhaled corticosteroid dosing for maintenance treatment of asthma in pediatric patients. J Asthma.

[70] Luks V, Burkett A, Turner L, Pakhale S (2013). Effect of physical training on airway inflammation in animal models of asthma: a systematic review. BMC Pulm Med.

[71] Alberca-Custódio RW, Greiffo FR, MacKenzie B, Oliveira-Junior MC, Andrade-Sousa AS, Graudenz GS, Santos AB, Damaceno-Rodrigues NR, Castro-Faria-Neto HC, Arantes-Costa FM, Martins Mde A, Abbasi A, Lin CJ, Idzko M, Ligeiro Oliveira AP, Northoff H, Vieira RP (2016). Aerobic exercise reduces asthma phenotype by modulation of the leukotriene pathway. Front Immunol.

[72] Manion AB, Velsor-Friedrich B (2017). Quality of life and health outcomes in overweight and non-overweight children with asthma. J Pediatr Health Care.

[73] Kalyva E, Eiser C, Papathanasiou A (2016). Health-related quality of life of children with asthma: self and parental perceptions. Int J Behav Med.

[74] Kanemitsu Y, Niimi A, Matsumoto H, Iwata T, Ito I, Oguma T, Inoue H, Tajiri T, Nagasaki T, Izuhara Y, Petrova G, Birring SS, Mishima M (2016). Gastroesophageal dysmotility is associated with the impairment of cough-specific quality of life in patients with cough variant asthma. Allergol Int.

[75] Favreau H, Bacon SL, Labrecque M, Lavoie KL (2014). Prospective impact of panic disorder and panic-anxiety on asthma control, health service use, and quality of life in adult patients with asthma over a 4-year follow-up. Psychosom Med.

[76] Krouse HJ, Yarandi H, McIntosh J, Cowen C, Selim V (2008). Assessing sleep quality and daytime wakefulness in asthma using wrist actigraphy. J Asthma.

[77] Luyster FS, Teodorescu M, Bleecker E, Busse W, Calhoun W, Castro M, Chung KF, Erzurum S, Israel E, Strollo PJ, Wenzel SE (2012). Sleep quality and asthma control and quality of life in non-severe and severe asthma. Sleep Breath.

[78] Schauer U, Bergmann KC, Gerstlauer M, Lehmann S, Gappa M, Brenneken A, Schulz C, Ahrens P, Schreiber J, Wittmann M, Hamelmann E, all members of the German Asthma Net (GAN). Improved asthma control in patients with severe, persistent allergic asthma after 12 months of nightly temperature-controlled laminar airflow: an observational study with retrospective comparisons. Eur Clin Respir J. 2015:2. 10.3402/ecrj.v2.28531 PMC4629753 26557252

[79] Pretolani M, Bergqvist A, Thabut G, Dombret MC, Knapp D, Hamidi F, Alavoine L, Taillé C, Chanez P, Erjefält JS, Aubier M. Effectiveness of bronchial thermoplasty in patients with severe refractory asthma: Clinical and histopathologic correlations. J Allergy Clin Immunol. 2016;5 [Epub ahead of print] 10.1016/j.jaci.2016.08.009 27609656

[80] Torrego A, Solà I, Munoz AM, Roqué I, Figuls M, Yepes-Nuñez JJ, Alonso-Coello P, Plaza V (2014). Bronchial thermoplasty for moderate or severe persistent asthma in adults. Cochrane Database Syst Rev.

